# Angiotensin II type 1 receptor-associated protein deficiency attenuates sirtuin1 expression in an immortalised human renal proximal tubule cell line

**DOI:** 10.1038/s41598-019-52566-y

**Published:** 2019-11-12

**Authors:** Takahiro Yamaji, Akio Yamashita, Hiromichi Wakui, Kengo Azushima, Kazushi Uneda, Yumiko Fujikawa, Sona Haku, Ryu Kobayashi, Kohji Ohki, Kotaro Haruhara, Sho Kinguchi, Takeo Ishii, Takayuki Yamada, Shingo Urate, Toru Suzuki, Eriko Abe, Shohei Tanaka, Daisuke Kamimura, Tomoaki Ishigami, Yoshiyuki Toya, Hidehisa Takahashi, Kouichi Tamura

**Affiliations:** 1 Department of Medical Science and Cardiorenal Medicine, Yokohama City University Graduate School of Medicine, Yokohama, Japan; 2 Department of Molecular Biology, Yokohama City University Graduate School of Medicine, Yokohama, Japan; 3 Cardiovascular and Metabolic Disorders Program, Duke-NUS Medical School, Singapore, Singapore; 4 Department of Medicine, Mount Sinai Beth Israel, New York, New York USA

**Keywords:** Chronic kidney disease, Cell biology

## Abstract

The proximal tubule is a particularly important site for ageing-related kidney damage. Sirtuin 1 (SIRT1), an NAD^+^ (nicotinamide adenine dinucleotide)-dependent deacetylase in the proximal tubule, may be involved in renal injury associated with ageing. However, the mechanisms of SIRT1 regulation remain to be elucidated. We recently reported that angiotensin II type 1 receptor (AT1R)-associated protein (ATRAP)-deficient mice displayed age-associated renal function decline and tubulointerstitial fibrosis. Our data showed that SIRT1 protein expression was reduced in ATRAP-deficient mice, although the relationship between ATRAP deficiency and age-associated renal fibrosis is still not fully understood. It is, therefore, necessary to investigate how ATRAP affects SIRT1 protein expression to resolve ageing-associated kidney dysfunction. Here, since ageing studies are inherently lengthy, we used an *ex vivo* model of the proximal tubule to determine the role of ATRAP in SIRT1 protein expression. We first generated a clonal immortalised human renal proximal tubule epithelial cell line (ciRPTEC) expressing AT1R and ATRAP. Using this cell line, we demonstrated that ATRAP knockdown reduced SIRT1 protein expression in the ciRPTEC but did not alter *SIRT1* mRNA expression. Thus, ATRAP likely mediates SIRT1 protein abundance in ciRPTEC.

## Introduction

The kidney is readily affected by ageing-associated changes, including glomerulosclerosis, tubular atrophy and interstitial fibrosis^1^. In particular, renal tubulointerstitial fibrosis is a final common pathway in most forms of progressive renal disease^2,3^.

Several studies have proven that kidney fibrosis accelerates with ageing and causes renal function decline^4,5^. The most common pathological change in patients with chronic kidney disease is fibrosis in the renal interstitium, and the severity of interstitial fibrosis is known to correlate with renal function decline^6^.

Several mechanisms causing kidney ageing are claimed; one of the most well-known is the free radical theory. In this theory, mitochondria play an important role in metabolizing reactive oxygen species (ROS)^7–9^. Decreased mitochondrial biogenesis due to ageing causes reduced levels of sirtuin 1 protein (SIRT1).

SIRT1 is a member of the nicotinamide adenine dinucleotide (NAD^+^)-dependent deacetylase family of proteins that neutralise ageing-related functional changes^10,11^. Aging decreases tissue NAD^+^ levels and SIRT1 activity, causing increased ROS formation in the mitochondria^12^. The SIRT1 protein is highly expressed in the renal tubules and is closely involved in renal physiology and pathology^13–16^. Various factors regulate SIRT1 expression. At the cellular level, the expression level of SIRT1 is maintained by protein and mRNA stability^17^. In addition, various systemic factors affect SIRT1 expression; for example, serum or nutrient starvation alters the expression level of SIRT1 in cells^18–20^.

Several different cell types contribute to renal tubulointerstitial fibrosis, including tubular epithelial cells, myofibroblasts, endothelial cells and inflammatory cells. In particular, the proximal tubule plays an important role in kidney function by maintaining the homeostasis of body salt and fluid, and it is also a very important site of ageing-related kidney damage^21^. Hence, evaluating the regulation of SIRT1 expression using proximal tubule cells could help to clarify the pathology of transporter-related renal disorders involved in electrolyte migration.

Angiotensin II type 1 receptor (AT1R)-associated protein (ATRAP) has been identified as a protein that binds specifically to the carboxyl-terminal domain of AT1R^22^. We have shown that ATRAP functions as an endogenous inhibitor that suppresses AT1R hyperactivation at local tissue sites^23–26^. Endogenous ATRAP is most abundantly expressed in the kidney, especially in proximal tubule epithelial cells. We recently demonstrated *in vivo* that ATRAP deficiency exacerbates ageing-associated renal function decline and tubulointerstitial fibrosis in systemic ATRAP knockout mice^27^. As a key mechanism, renal SIRT1 expression was significantly decreased in the aged ATRAP-knockout mice compared to the aged wild-type mice, possibly in an angiotensin-independent manner. However, the mechanisms by which ATRAP regulates SIRT1 expression in the renal proximal tubules has not yet been defined. Therefore, in the present study, we aimed to reveal the regulatory function of ATRAP with regards to SIRT1 expression using a clonal immortalised human renal proximal tubule epithelial cell line (ciRPTEC). We demonstrated that ATRAP plays a role in the regulation of SIRT1 protein levels but not that of *SIRT1* mRNA levels in ciRPTEC.

## Results

### A clonal immortalised renal proximal tubule epithelial cell line expressing AT1R and ATRAP and reacting to angiotensin II

**Figure 1:**
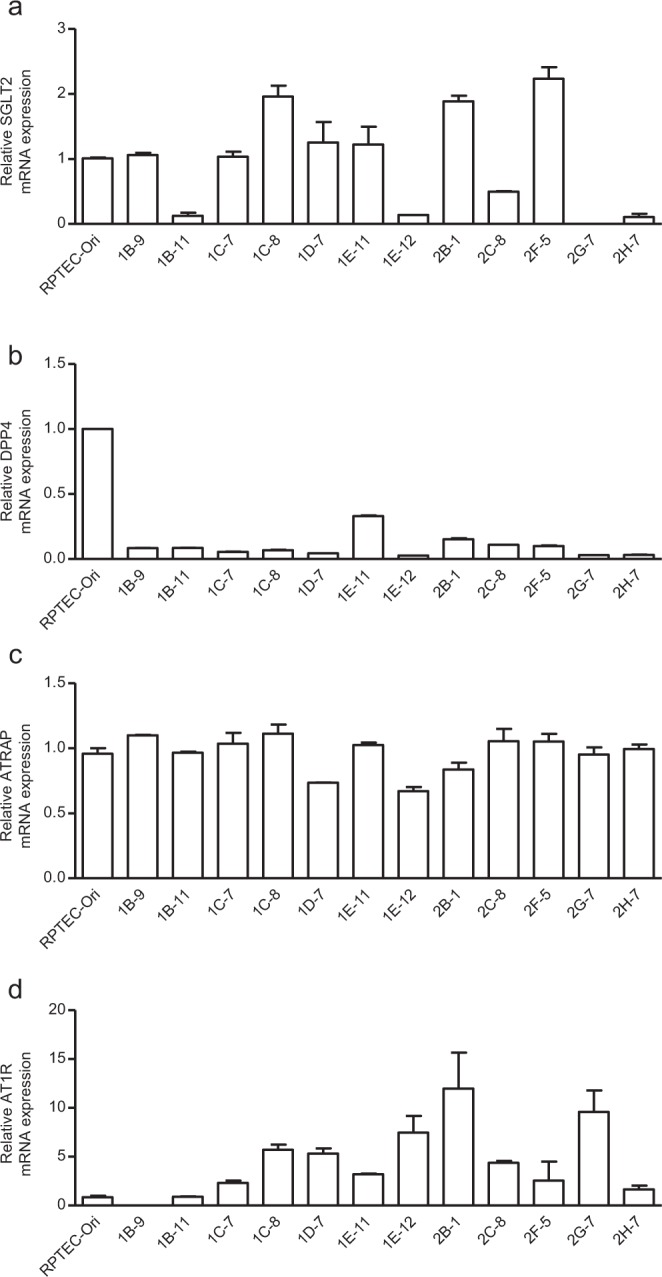
mRNA expression of the proximal tubule markers, *AT1R* and *ATRAP*, in clonal immortalised cells. (**a–d**) The relative mRNA levels of *SGLT2*, *DPP4*, *ATRAP* and *AT1R* in 12 clonal immortalized cell (ciRPTEC) clones were determined by RT-qPCR, normalized to 18S ribosomal RNA. The mRNA levels of the original RPTEC (RPTEC-Ori) were set to 1. Data were obtained with three biologically independent experiments. Values represent the means ± standard error.

To analyse the function of ATRAP in human proximal tubule cells, we produced an immortalised RPTEC line by expressing human Telomerase Reverse Transcriptase (hTERT) and small hairpin RNA (shRNA)-targeted CDKN2A. Then, we cloned the immortalised RPTEC and characterised the cells based on the expression of two proximal tubule markers, SGLT2^28,29^ and DPP4^30^. Among the 12 cell clones obtained, clones 1C-8, 2B-1 and 2F-5 showed high mRNA expression of *SGLT2* (Fig. 1a). Among these three clones, clone 2B1 showed the highest mRNA expression of *DPP4*, another proximal tubule marker that is expressed by human primary RPTEC (Fig. 1b). We next analysed *AT1R* and *ATRAP* mRNA expression in the ciRPTEC clones. All 12 clones maintained *ATRAP* expression (Fig. 1c) and clone 2B1 showed the highest *AT1R* expression (Fig. 1d). We further confirmed the protein expression of SGLT2 and DPP4 by immunofluorescence staining and the expression of ZO-1, an epithelial marker, was also observed (Supplementary Fig. S1). We also observed the cell morphology of ciRPTEC_2B1 with phase contrast microscopy (Supplementary Fig. S1). The results for SGLT2 and DPP4 were further validated by western blotting (Supplementary Fig. S2). Based on these results, we selected clone ciRPTEC_2B1 for further analysis.

**Figure 2:**
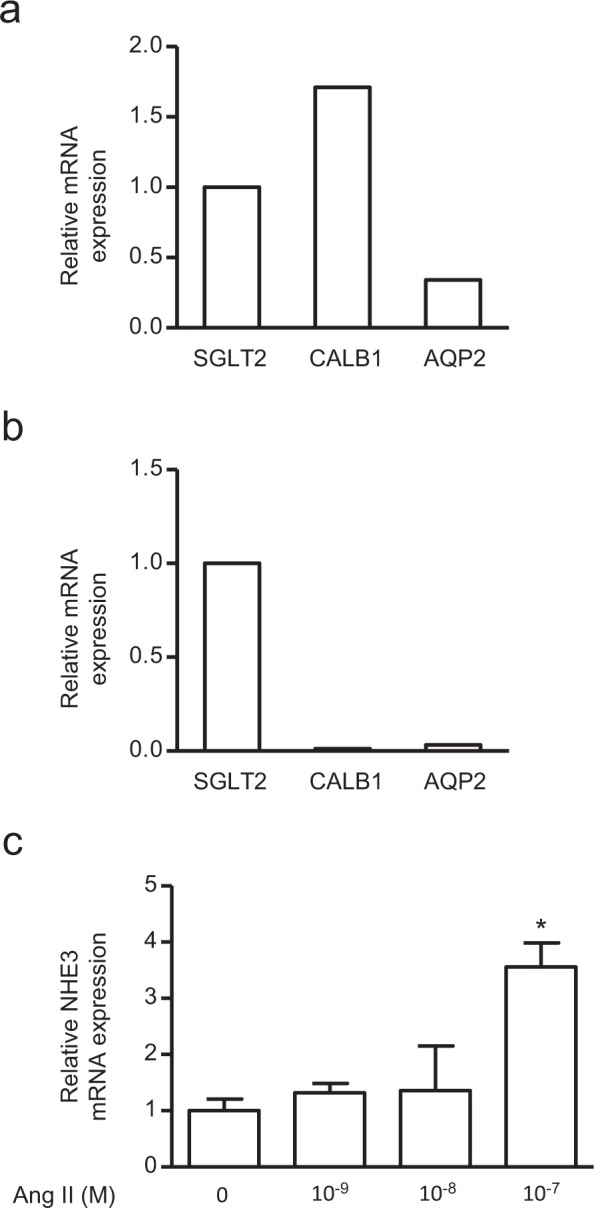
Comparison of mRNA expression levels of distal and proximal tubule markers in the ciRPTEC 2B-1 cell line, and reactivity of NHE3 in this cell line to angiotensin II (Ang II) treatment. (**a,b**) The relative mRNA levels of *CALB1* and *AQP2* in the original RPTEC (RPTEC-Ori) cell line and the clonal immortalized cell line 2B1 (ciRPTEC 2B1) were determined by RT-qPCR, normalized to 18S ribosomal RNA. mRNA levels of *SGLT2* were set to 1. (**c**) The relative mRNA levels of *NHE3* in ciRPTEC 2B1 after 24 hours of treatment with a range of Ang II concentrations were determined by RT-qPCR, normalized to 18S ribosomal RNA. mRNA levels obtained without Ang II (concentration 0 M) were set to 1. Data were obtained with three biologically independent experiments. Values represent the means ± standard error. *p < 0.05 vs. Ang II 0 M group. Data were analysed by one-way ANOVA.

Our original human primary RPTEC previously reported expressed both renal proximal (*SGLT2*) and distal tubule (*CALB1*^31^ and *AQP2*^32,33^) markers indicating possible contamination with distal tubule cells (Fig. 2a). We, therefore, used RT-qPCR to verify the mRNA expression of these markers in ciRPTEC_2B1. Compared with the proximal tubule marker, *SGLT2*, low mRNA expression of the distal renal tubule markers, *CALB1* and *AQP2*, was observed in ciRPTEC_2B1 compared with the original RPTEC (Fig. 2b).

The renal proximal tubule is suggested to be involved in Ang II-mediated hypertension^34^ and fibrosis^35–37^. Since the Na^+^/H^+^ exchanger-3 (NHE3) is primarily responsible for maintaining the balance of sodium, Ang II infusion enhances the expression of NHE3 in the proximal tubule in various organisms^38–40^. To further characterise our ciRPTEC_2B1, we examined the mRNA expression of *NHE3* following Ang II treatment. The results showed that 10^−9^ to 10^−7^ M of the Ang II treatment stimulated *NHE3* mRNA expression in a dose-dependent manner (Fig. 2c). These results indicated that ciRPTEC_2B1 is an immortalised renal proximal tubule cell line expressing NHE3 and that it is competent for Ang II stimulation.

These observations suggested that ciRPTEC_2B1 is a cell line with the properties of RPTECs and we renamed this cell line as ciRPTEC for further reference.

### Gene expression regulation of *ATRAP* and *SIRT1* in ciRPTEC

**Figure 3:**
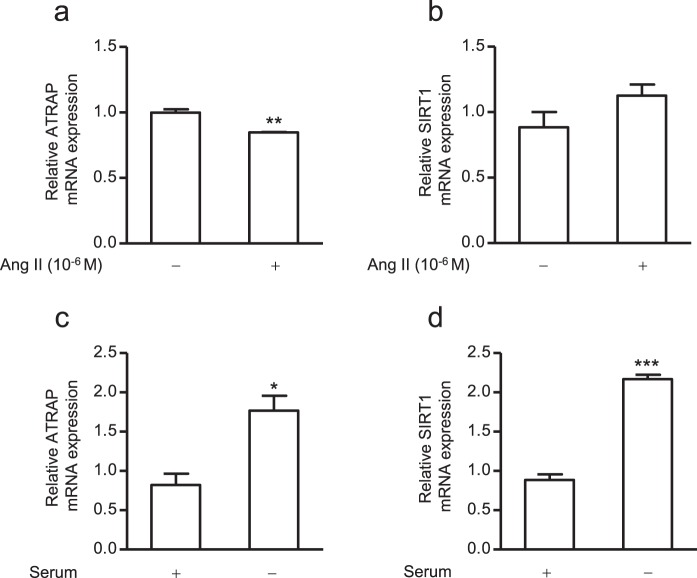
mRNA expression levels of *ATRAP* and *SIRT1* in ciRPTEC in response to angiotensin II (Ang II) treatment or serum withdrawal. The ciRPTEC were treated with 10^−6^ M of Ang II (+) for 24 hours (**a,b**) or serum withdrawal (−) for 24 hours (**c,d**). The relative mRNA levels of *ATRAP* and *SIRT1* in the ciRPTEC were determined by RT-qPCR, normalized to 18 S ribosomal RNA. mRNA levels in the absence of Ang II treatment (−; for **a,b**) or presence of serum (+; for **c,d**) were set to 1. All data were obtained with three biologically independent experiments. Values represent the means ± standard error. (**a**) **p < 0.01 vs. Ang II non-stimulation group. (**c,d**) *p < 0.05, ***p < 0.001 vs. normal serum group. Data were analysed with the unpaired Student’s t-test.

We recently demonstrated that ageing-associated kidney fibrosis was exacerbated in ATRAP-knockout mice^27^. In addition, Sirt1 protein, which plays a role in various ageing-related changes, was decreased in both the renal proximal tubules and distal tubules in aged ATRAP-knockout mice kidneys^27^; in contrast, *Sirt1* mRNA expression did not show this tendency. Since the origin of ATRAP knockout-dependent reductions in Sirt1 protein have not been elucidated for the kidney, we analysed gene expression regulation of ATRAP and SIRT1 in ciRPTEC. For this purpose, we determined the effect of Ang II on the mRNA expression of *ATRAP* and *SIRT1* in ciRPTEC, since the administration of Ang II has been shown to reduce *ATRAP* but not *SIRT1* mRNA expression in the mouse kidney^27,41^. In ciRPTEC, Ang II treatment significantly reduced the *ATRAP* mRNA level (Fig. 3a). However, no significant alteration was observed in the *SIRT1* mRNA level following Ang II treatment (Fig. 3b).

We next investigated the effect of serum starvation on the expression levels of *ATRAP* and *SIRT1* mRNA in ciRPTEC. Both *ATRAP* and *SIRT1* mRNA levels have been shown to increase following serum starvation in mDCT (mouse distal convoluted tubule; for *ATRAP* mRNA)^42^ or HEK293 and HeLa (for *SIRT1* mRNA) cells^19^. Our findings showed that accumulation of *ATRAP* and *SIRT1* mRNA was significantly stimulated by serum starvation in ciRPTEC (Fig. 3c,d).

Although *in vivo* mouse ageing and *in vitro* serum starvation of cells are not closely related stimuli, the above results prompted us to use ciRPTEC for *in vitro* functional analysis of ATRAP in the expression regulation of SIRT1 in response to serum starvation.

### Effect of ATRAP knockdown and knockout on SIRT1 expression in ciRPTEC

**Figure 4:**
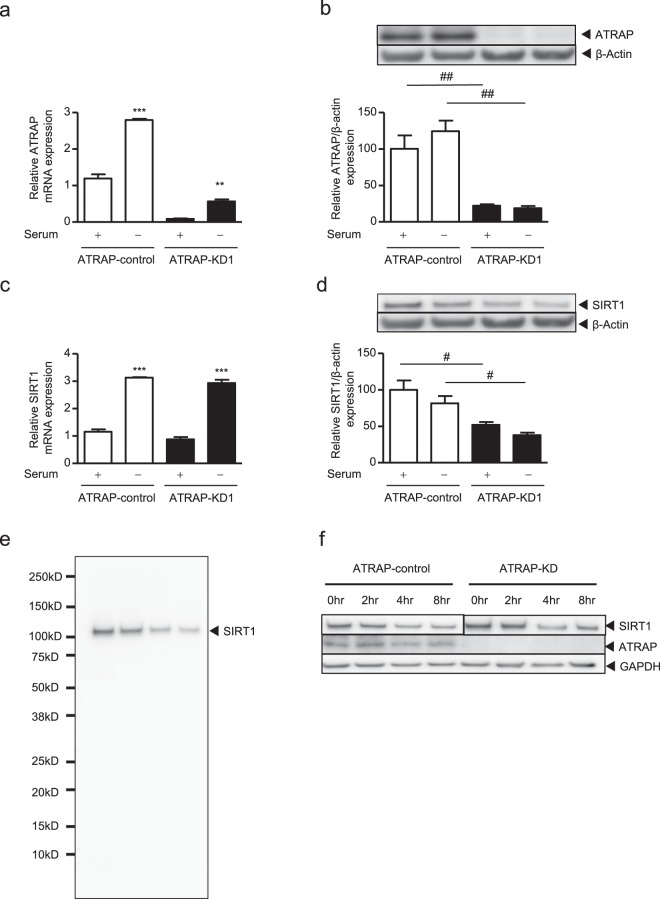
Effect of ATRAP knockdown on *SIRT1* mRNA and protein expression with or without serum-withdrawal. The ciRPTEC were treated with negative control siRNA (ATRAP-control, **a–f**), ATRAP siRNA #1 (ATRAP-KD1, **a–f**) for 48 hours, followed by serum withdrawal for 24 hours (**a–e**). (**a,c**) The relative mRNA levels of *ATRAP* (**a**) or *SIRT1* (**c**) on ciRPTEC were determined by RT-qPCR, normalized to 18 S ribosomal RNA. mRNA levels in the presence of serum (+) and the control siRNA were set to 1. (**b,d**) The relative protein expression of ATRAP and SIRT1 in the ciRPTEC was determined by western blot analysis, normalized to β-actin expression. Protein levels in the presence of serum (+) and control siRNA were set to 100. SIRT1 proteins were detected with an antibody towards the N-terminal 1–131 amino acids (SIRT1_N lot 2465249). (**e**) SIRT1 protein detected with an antibody recognising the C-terminal region (SIRT1_C). (**f**) Half-life analysis of the SIRT1 protein was performed 48 hours after siRNA transfection of ciRPTEC cells and treatment with emetine to repress de-novo protein synthesis. Cell lysates were collected at 0, 2, 4 and 8 hours after emetine treatment. SIRT1 proteins were detected with the SIRT1_C antibody. Since the SIRT1 expression level at time 0 was decreased in ATRAP-KD1, the signal intensities of the SIRT1 proteins at 0 hours were set to similar levels visually between the ATRAP-control and ATRAP-KD1 by showing the short exposure (ATRAP-control) and the long exposure (ATRAP-KD1) images. Original gel images are presented in Supplementary Fig. S12. All data were obtained with three biologically independent experiments (except for (**h**) where two replicates were used) and were analysed by two-way ANOVA. Values represent the means ± standard error. (**a,c**) **p < 0.01 and ***p < 0.001 vs. serum (+) within the same ATRAP groups. (**b,d**) ^#^p < 0.05 and ^##^p < 0.01 vs. ATRAP-control within the same serum groups.

We determined the effect of siRNA-mediated *ATRAP* knockdown in ciRPTEC. The results indicated that the siRNA targeting human *ATRAP* led to diminished mRNA and protein expression of ATRAP in ciRPTEC (Fig. 4a,b).

*SIRT1* mRNA expression, which was induced by serum starvation, was unaffected by transient ATRAP knockdown (Fig. 4c). On the other hand, SIRT1 protein expression as detected with two SIRT1 antibodies recognising the N-terminal 131 amino acids (SIRT1_N lots 2465249 and 3104232) was not induced by serum starvation in our ciRPTEC cells, although transient ATRAP knockdown reduced the expression of SIRT1 protein under both normal and serum-starved conditions (Fig. 4d and Supplementary Fig. S3). To evaluate the production of SIRT1 protein isoforms encoded by alternative splice/transcriptional start site mRNAs^43^, we used the SIRT1 antibody recognising the C-terminal region (SIRT1_C) as a western blotting probe; similar results were obtained to those with the SIRT1_N antibodies and no obvious alternative isoforms of the SIRT1 protein were detected (Fig. 4e and Supplementary Fig. S3).

To exclude any off-target effect of ATRAP siRNA #1, we repeated all the siRNA-based experiments with a siRNA targeted towards a different site along the human *ATRAP* mRNA (ATRAP siRNA #2). Similar results were obtained, with ATRAP knockdown-induced reduction of SIRT1 protein observed when using ATRAP siRNA #2 (Supplementary Fig. S4a,b).

Since SIRT1 protein abundance can be regulated through mechanisms affecting protein stability^44^, we examined the SIRT1 protein half-life using the protein synthesis inhibitor, emetine^45^. The results showed that transient knockdown of ATRAP did not destabilize SIRT1 protein in the ciRPTEC cells (Fig. 4f).

**Figure 5:**
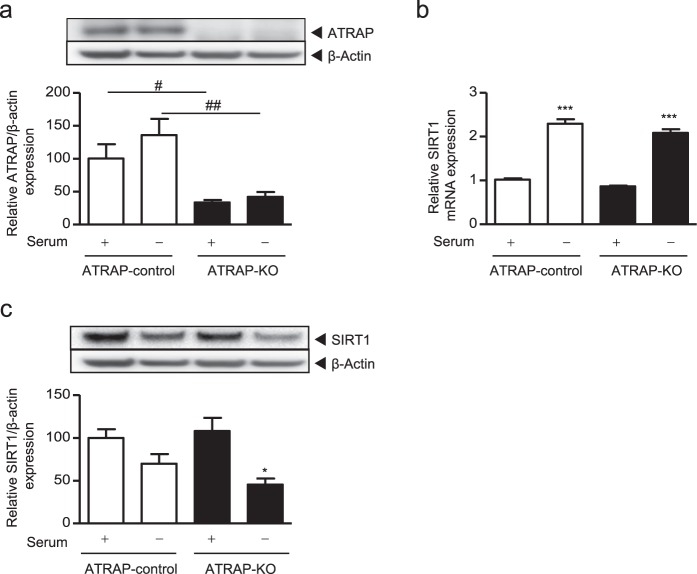
Effect of ATRAP knockout generated using CRISPR-CAS9 on *SIRT1* mRNA and protein expression levels under serum-starvation. ATRAP-KO (ciRPTEC expressing CAS9 and gRNA targeted towards ATRAP) or ATRAP-control (ciRPTEC expressing only CAS9) cells were cultured with or without serum for 24 hours. (**a,c**) The relative protein expression of ATRAP and SIRT1 in the ciRPTEC was determined by western blot analysis, normalized to β-actin expression. Protein levels of ATRAP-control with serum (+) were set to 100. (**b**) The relative mRNA levels of *SIRT1* in the ciRPTEC was determined by RT-qPCR, normalized to 18 S ribosomal RNA. The mRNA level of ATRAP-control with serum (+) was set to 1. All data were obtained with three biologically independent experiments. Values represent the means ± standard error. All data were analysed by two-way ANOVA. (**a**) ^#^p < 0.05 and ^##^p < 0.01 vs. ATRAP-control within the same serum groups. (**b**) ***p < 0.001 vs. serum (+) within the same ATRAP groups. (**c**) *p < 0.05 vs. serum (+) within the same ATRAP groups.

Because transient knockdown cells still retained a considerable amount of *ATRAP* mRNA, especially under serum-starved conditions, we further analysed the effect of stable ATRAP knockout on SIRT1 protein expression using the CRISPR-CAS9 system^46^. For this purpose, we established ATRAP knockout ciRPTEC and control ciRPTEC cells by infecting the cells with lentivirus expressing CAS9 and Puro^R^, and either a single guide RNA (sgRNA) targeting the human *ATRAP* gene or non-targeted sgRNA. Infected cells were selected for with puromycin, and ATRAP knockout in ciRPTEC was confirmed by western blotting. (Fig. 5a). ATRAP knockout did not affect *SIRT1* mRNA expression under either the normal or serum-starved condition (Fig. 5b). However, SIRT1 protein expression was significantly decreased by serum starvation in ATRAP knockout cells, while no significant reduction in SIRT1 protein was observed in the control cells (Fig. 5c).

Taken together, these results indicated that ATRAP may be one of the molecules involved in regulating the abundance of SIRT1 protein but not *SIRT1* mRNA.

## Discussion

In the present study, we demonstrated that ATRAP depletion reduced SIRT1 protein levels only and not *SIRT*1 mRNA levels in a newly developed ciRPTEC. Therefore, our results indicated that proximal tubule ATRAP can regulate SIRT1 expression. Intriguingly, ATRAP deficiency did not alter *SIRT1* mRNA expression either in the presence or absence of serum starvation; even though serum starvation alone induced *SIRT1* mRNA accumulation, SIRT1 protein levels were unaltered in the ciRPTEC. In addition, ATRAP knockdown did not affect SIRT1 protein stability. These results suggested that SIRT1 protein abundance in ciRPTEC may be regulated post transcription but before the level of protein synthesis. Consistent with this view, SIRT1 translation is regulated by microRNAs, which can repress the translation of partially complementary mRNAs^47^. In addition to microRNAs, other uncharacterized RNA binding proteins may regulate SIRT1 mRNA translation. For example, HuR, an AU-rich element-binding protein, binds to and stabilizes SIRT1 mRNA, while other AU-rich element-binding proteins have the ability to repress translation (e.g. CUGBP2 and TIA1/TIAL1), making these proteins candidates for SIRT1 regulation^47–49^. Another possibility of post-transcriptional regulation of SIRT1 expression is the splicing regulation of SIRT1 pre-mRNA. Since we analysed mRNA abundance with the RT-qPCR method (where the probe targeted the exon junction of exons 8 and 9 of RefSeq: NM_012238), we should not exclude the possibility of uncharacterized and unproductive alternative splicing products of SIRT1 pre-mRNA. In support of this theory, HuR and TIA1 (also known as TIAL1) positively and negatively regulate the inclusion of SIRT1 exon 8, respectively^43^, and CUGBP2 negatively regulates the exclusion of SIRT1 exons 2–9 and the inclusion of a new exon^50^. Based on the western blotting observations made when using SIRT1 antibodies against the N-terminal 1–131  amino acids region and the C-terminal region, we did not capture any SIRT1 protein isoforms encoded by known alternative transcriptional start sites (RefSeq: NM_001142498 and NM_001314049) or splice sites^43,50,51^ in the ATRAP knockdown condition. SIRT1 isoform schema is shown in Supplementary Figure (Supplementary Fig. S5). Future analyses are required to further define the mechanisms by which ATRAP regulates SIRT1 protein abundance in ciRPTEC. Although the models are different, it is notable that we observed ATRAP deficiency to reduce SIRT1 protein abundance in the aged mouse kidney^27^. It would be intriguing to resolve and compare the mechanisms of ATRAP regulation of SIRT1 in ciRPTEC and the mouse kidney.

As described above, the *ex vivo* approach using immortalised differentiated cells can provide a suitable model for unravelling the detailed mechanisms of cellular ATRAP function. Since ATRAP is expressed and has activity in various tissues including the proximal tubule, distal tubule, aorta, adipose tissue and muscle^23,26,52,53^, similar analyses in these tissues should also prove beneficial in resolving the detailed mechanisms of action of ATRAP.

Our ciRPTEC line was immortalised by hTERT with p16 knockdown and expressed high levels of not only a proximal marker gene but also AT1R. Furthermore, the reactivity of NHE3 dependent on Ang II was also confirmed. Although RPTEC/TERT1 cells are an immortalised RPTEC cell line prepared using only TERT1 and are commercially available, this cell line has not been characterised regarding AT1R signalling^54,55^. Hence, our ciRPTEC will provide an alternative resource for immortalised RPTEC and could be used to analyse other proximal tubule-related proteins in addition to ATRAP.

## Methods

### Establishment of the clonal immortalised renal proximal tubule epithelial cell (ciRPTEC) line and the ATRAP knockout

Normal human renal proximal tubule epithelial cells (RPTEC) were purchased from Lonza (#CLCC-2553, lot 0000203150, Caucasian female, 10 years old). Normal human RPTEC cells were immortalised by infection with lentivirus expressing hTERT and short hairpin RNA (shRNA) targeting p16 (plenti6_TERT_sh-p16).

To generate plenti6_TERT_sh-p16 (siRNA sequence targeted towards CDKN2A along nucleotides 419–437 of NM_000077: CCAACGCACCGAATAGTTA), we inserted a U6 promoter-driven shRNA expression cassette at the NotI site of pLenti6_V5/DEST (Thermo Fisher Scientific). We constructed pENTR-A_TERT, then inserted it into the pLenti6_V5/DEST_sh-p16 vector using the Gateway cloning system (Thermo Fisher Scientific).

We generated the pL-CRISPR-EFh-puro^R^_sg-AGTRAP plasmid (containing a single guide RNA sequence targeted towards AGTRAP: TAGAGCAGGACTTACCGGGG) using a standard method. The production of the lentiviral supernatant was performed as follows. The day before transfection (Day 1), 6 × 10^6^ 293FT cells were seeded in a poly-L-lysine-coated 10-cm tissue culture plate. On the day of transfection (Day 2), we prepared a DNA/polyethyleneimine (PEI) mixture. In brief, 10.8 µg of pLenti_sh-p16_hTERT, pL-CRISPR-EFh-puro^R^_sg-AGTRAP or pL-CRISPR-EFh-puro^R^ (no insert), 12.15 µg of ViraPower Lentiviral Packaging Mix (Thermo Fisher Scientific) and 2.55 µg of the pAdVantage vector (Promega) were mixed in 800 µL of Opti-MEM I medium (Thermo Fisher Scientific) and added to 51 µg of PEI (1 mg/mL, Polyethylenimine “Max”; Polysciences) in 800 µL of Opti-MEM I, followed by mixing and incubation for 30 min at room temperature. During incubation of the DNA/PEI mixture, we removed the culture medium from the 293FT cells and replaced it with 5 mL of Opti-MEM I medium containing 10% foetal bovine serum (FBS). After incubation, the DNA-PEI mixtures were added to the 293FT cells, which were then cultured for 8 h. The medium was exchanged for Dulbecco’s modified Eagle’s medium (DMEM) containing 10% FBS and 10 µM forskolin and the cells were cultured for 24 h. The culture supernatants were collected and filtered with a 0.22-µm Steriflip filter (Millipore) to generate the lentiviral supernatant.

For lentiviral infection, 5 mL of the lentiviral supernatants were incubated with primary human RPTEC cells for 24 h at 37 °C in a 5% CO_2_ incubator, then the lentiviral supernatants were discarded followed by the addition of DMEM. For immortalisation, cells were cultured for more than 59 PDL. A non-infected control was cultured for the primary human RPTEC cells as a cellular senescence control. Immortalised RPTEC (iRPTEC) cells were cloned with limiting dilution and 12 clones were obtained. Cloned iRPTEC (ciRPTEC) and all other RPTEC cells were maintained in REGM medium (Lonza).

For the ATRAP knockout, ciRPTEC clone 2B1 cells were infected with lentivirus expressing CAS9, puromycin N-acetyltransferase (Puro^R^) and either a sgRNA targeted towards AGTRAP (ATRAP-KO) or a non-targeted sgRNA (control). Cells were selected with 2 µg/mL of puromycin. Puromycin-selected heterogeneous ciRPTEC_ATRAP-KO or ciRPTEC_control were used. pL-CRISPR-EFh-puro^R^ was constructed from pL-CRISPR-EFh-GFP-BSD, which was obtained from Dr Yoshinori Sato at the Yokohama City University School of Medicine. For this purpose, sgRNA targeting exon 4 of ATRAP (ATRAP: AGGTGGTGGCCTCACCAGTGTGG, underlined: PAM sequence) was designed with the CHOPCHOP tool and used^56^. The detailed sequence will be provided upon request.

### siRNA and transfection

The following siRNA sequences designed by i-Score^57^ were used: ATRAP siRNA #1: UACGGUCCUGAGAAGACCC and ATRAP siRNA #2: GGGUCUUCUUAGGAUCGUG. For the non-silencing control, AllStars Negative Control siRNA (Qiagen) was used. Since ATRAP siRNA #1 had higher knockdown efficiency than ATRAP siRNA #2, we used ATRAP siRNA #1 in most experiments.

siRNA transfections were performed in 6-well plates with Lipofectamine RNAiMax Reagent (Thermo Fisher Scientific) according to the manufacturer’s protocol, and cells were harvested 48 h later.

### Reverse transcription quantitative polymerase chain reaction (RT-qPCR) analysis

Total RNA was extracted from the RPTEC with the NucleoSpin RNA Plus kit (Macherey Nagel), and cDNA was produced with the SuperScript III First-Strand Synthesis System (Invitrogen). RT-qPCR was performed with a Bio-Rad CFX96 Touch Real-Time PCR Detection System by incubating the reverse transcript product with TaqMan PCR Master Mix and a designed TaqMan probe (Applied Biosystems), essentially as described previously^23,52,53^. We used the following TaqMan probes: SIRT1 (Hs-01009006_m1), ATRAP (Hs01564425_m1), AT1R (Hs00258938_m1), NHE3 (Hs0090384_m1), SGLT2 (Hs01009006_m1), DPP4 (Hs00897391_m1), Calbindin2 (Hs01077197_m1) and AQP2 (Hs00292214_m1). The mRNA levels were normalised to those of the 18 S rRNA. RT-qPCR was performed for 50 cycles, and the expression of 18 S rRNA was confirmed at around 10 cycles, and at around 20–40 cycles for the target genes. The RQ value was calculated using the ΔΔCT method^58^. For angiotensin II treatment, recombinant angiotensin II was obtained from Sigma-Aldrich (#A9525, lot SLBT2492) to treat the cells for 24 h.

### Western blot analysis

Western blot analysis was performed as follows. Briefly, total protein was extracted from ciRPTEC with a sample buffer containing 1% sodium dodecyl sulphate. Then, the protein concentration of each sample was measured with a Qubit Protein Assay kit (Q33212, lot; 1985231 Thermo Fisher Scientific) using bovine serum albumin as the standard. An equal amount of each protein extract was resolved on a 5–20% polyacrylamide gel (Atto) and electrophoresed at 200 V for 37 min. After separation, proteins were transferred to a polyvinylidene fluoride (PVDF) membrane at 500 mA, 31 V for 15 min using an iBlot dry blotting system (Invitrogen). Membranes were blocked for over 1 h at room temperature with TBST containing 5% skim milk and probed overnight at 4 °C with specific primary antibodies towards SIRT1 (#07-131, lots 2465249 and 3104232 from Millipore; #2310 from Cell Signaling Technology), ATRAP (#ab85175; Abcam), GAPDH (#ab9485; Abcam) and β-actin (#A5441, Clone AC-15; Sigma-Aldrich). ATRAP antibody was diluted 1:1,000 with Signal Enhancer HIKARI for western blotting and ELISA (Nacalai Tesque), SIRT1 antibody was diluted 1:1,000 and the β-actin antibody was diluted 1:5,000 with the same solution.

Membranes were washed and further incubated with secondary antibodies for 60 min at room temperature. For ATRAP and SIRT1, the secondary antibody, ECL anti-rabbit IgG (#NA934-1ML, lot 9818407; GE Healthcare), was diluted 1:2,000 with TBST containing 5% skim milk, while HRP-conjugated anti-mouse IgG (#NA931-1ML, lot 399402; GE Healthcare) was diluted 1:5,000 for β-actin.

The sites of the antibody-antigen reaction were visualised with an enhanced chemiluminescence substrate (GE Healthcare). Images were analysed quantitatively with a Fujifilm LAS-4000 Image Analyser (Fujifilm).

Images of all membranes used in the Western blot are shown in Supplemental Figs 6–15.

### Half-life analysis of SIRT1 protein

ciRPTEC cells were transfected with the indicated siRNAs. Forty-eight hours later, the cells were treated with H_2_O (control) or 1 μg/mL emetine (#E-2375; Sigma-Aldrich) for the indicated periods and harvested. The total cell extracts were analysed by western blotting with the anti-SIRT1 and anti-GAPDH antibodies that were previously described.

### Statistical analysis

Statistical analyses were performed with GraphPad Prism software (GraphPad Software, La Jolla, CA, USA). All data are shown as the mean ± SEM. Differences were analysed using the following statistical tests. An unpaired t-test was used to analyse differences between two groups. Repeated-measures ANOVA was used to analyse differences over time. Two-way ANOVA with Bonferroni post hoc testing was used to test for differences following serum starvation stimulation within each genotype or differences between wild-type (WT) versus ATRAP-KO (knockout) or -KD (knockdown) cells. P values < 0.05 were considered statistically significant.

## Supplementary information

Supplement figure

## Data Availability

The data used to support the findings of this study are included in the article.
