# Supplementary material for: Angiotensin II type 1 receptor-associated protein deficiency attenuates sirtuin1 expression in an immortalised human renal proximal tubule cell line
Source: Sci Rep. 2019 Nov 12;9:16550. doi: 10.1038/s41598-019-52566-y (PMC6851135; doi:10.1038/s41598-019-52566-y)

Angiotensin II type 1 receptor-associated protein deficiency attenuates sirtuin 1 expression in an immortalised human renal proximal tubule cell line

Takahiro Yamaji, MD, Akio Yamashita, PhD, Hiromichi Wakui, MD, PhD, Kengo Azushima, MD, PhD, Kazushi Uneda, MD, PhD, Yumiko Fujikawa, DDS, PhD, Sona Haku, MD, Ryu Kobayashi, MD, PhD, Kohji Ohki, MD, PhD, Kotaro Haruhara, MD, PhD, Sho Kinguchi, MD, Takeo Ishii, MD, PhD,Takayuki Yamada, MD, Shingo Urate, MD, Toru Suzuki, MD, Eriko Abe, MD, Shohei Tanaka, MD, Daisuke Kamimura, MD, PhD, Tomoaki Ishigami, MD, PhD, Yoshiyuki Toya, MD, PhD, Hidehisa Takahashi, MD, PhD, Kouichi Tamura, MD, PhD.

Supplementary figures and methods

Supplemental Method S1

Immunofluorescence microscopy

1.0×10^5 of ciRPTEC cells were seeded onto collagen (IPC-50; AteloCell) coated 24 well dish with a 14mm across cover-slip (obtained from Matsuna). Twenty four hours after cell seeding, cells were fixed, permeabilized and blocked as follows. For SGLT2 and ZO-1, fixed with PBS-buffered 2% paraformaldehyde at room temperature for 15 minutes, permeabilized with 0.5% Triton X-100 at room temperature for 10 minutes and blocked with 5% normal goat serum (NGS) in PBS at room temperature for 1 hour. For DPP-4, fixed/permeabilized with cold methanol for 15 minutes at -20℃, and blocked as same way.

Primary antibodies were diluted at 1:200 (SGLT2 and DDP-4) or 1:500 (ZO-1) by a mixture of tris-buffered saline (TBS) and Polysorbate 20 (TBST) containing 0.1% BSA and 1.5% NGS and probed for 16 hours at 4oC. Alexa Fluor 488- (SGLT2 and DPP-4) and 555- (ZO-1) conjugated secondary antibodies (Molecular Probes) or 4,6-diamidino-2-phenylindole (DAPI), which stained genetic material, were used at 1:500 dilution in same buffer as primary antibodies for 1 hour at room temperature. Following antibodies/dye were used: DPP-4 (#67138; Cell Signaling Technology), SGLT2 (sc-393350; Santacruz), ZO-1(617300; Life technology) and DAPI (D9542; SIGMA). These cells were mounted with ProLong Gold (Life technology) and observed using a confocal microscope system (LSM 510; Carl Zeiss). Acquired images were analysed using Photoshop CS6 (Adobe Systems), according to the guidelines of the journal.

FigureS1

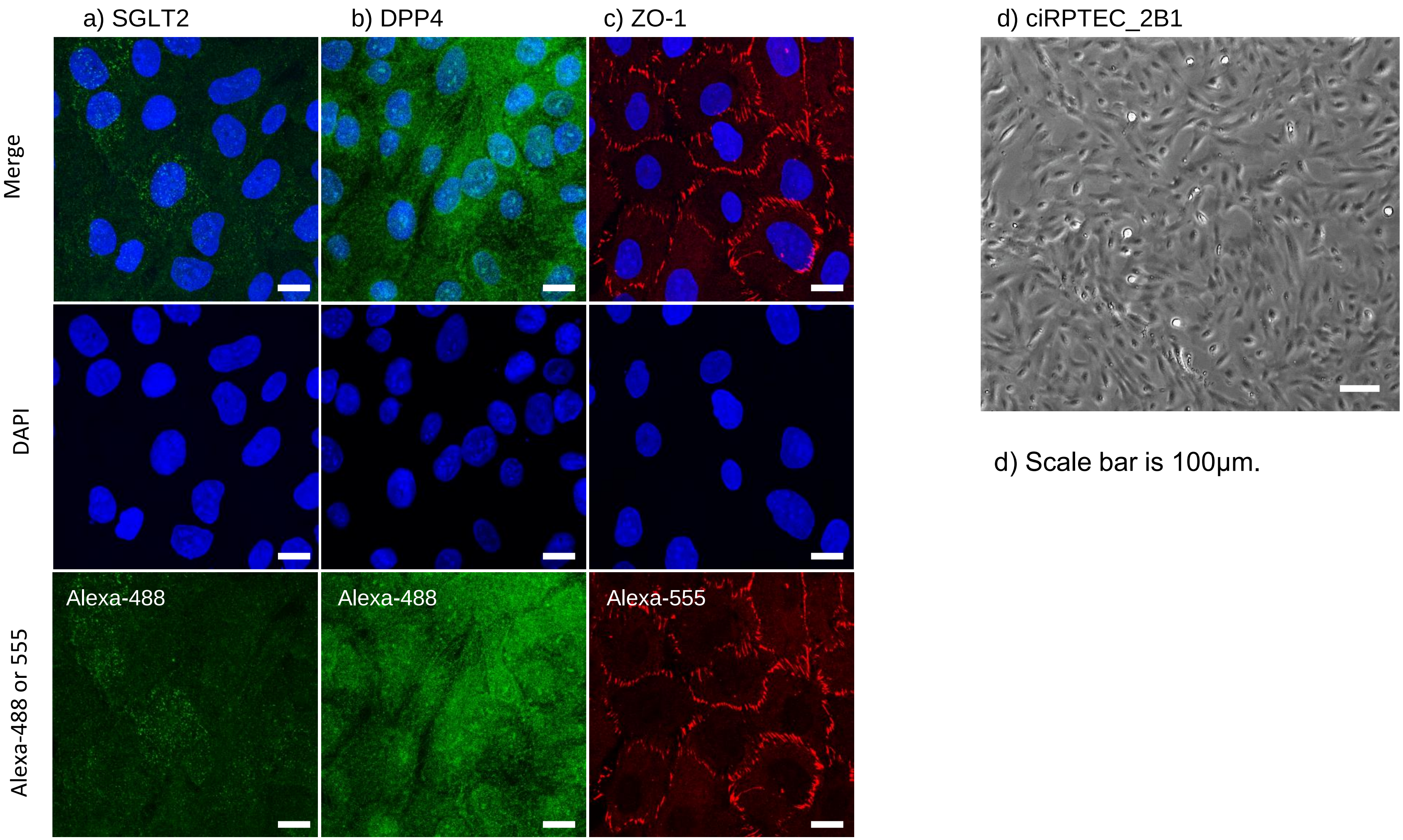

d) Scale bar is 100μm.

a-c) Scale bar is 20μm.

Supplemental Figure legend

FigureS1

Immunofluorescence analysis of protein expression of proximal tubule markers in ciRPTEC cell

a-c) ciRPTEC cells cultured on cover-slip for 2 days and immunostained with SGLT2, DPP4 and ZO-1. Representative images of confluent monolayers of ciRPTEC treated. A projected view of apical to basal sections is shown. Scale bars indicate 20 μm.

d) Transmission image of confluent ciRPTEC 2B1 cultured on normal dish. Phase contrast image was observed using EVOS FL system (Life technologies). Scale bars indicate 100 μm.

FigureS2

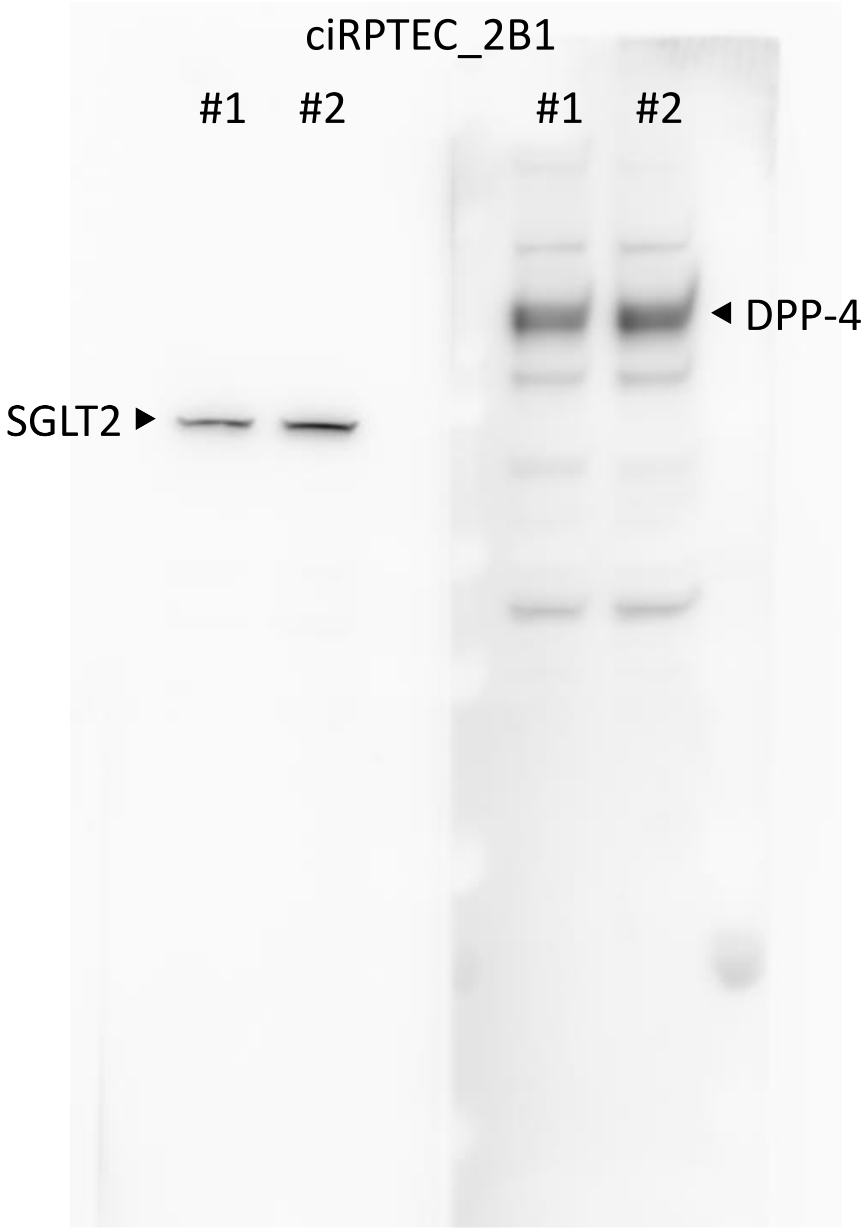

Supplemental Figure legend

FigureS2

Western blot analysis of protein expression of proximal tubule markers in ciRPTEC cell.

SGLT2 and DPP4 were probed and detected using same antibodies of Figure S1. Arrow heads indicated predicted molecular weight signals of indicated proteins.

1 and # 2 are ciRPTEC lysates collected on different days.

FigureS3

A whole gel of SIRT1 Figure 4e

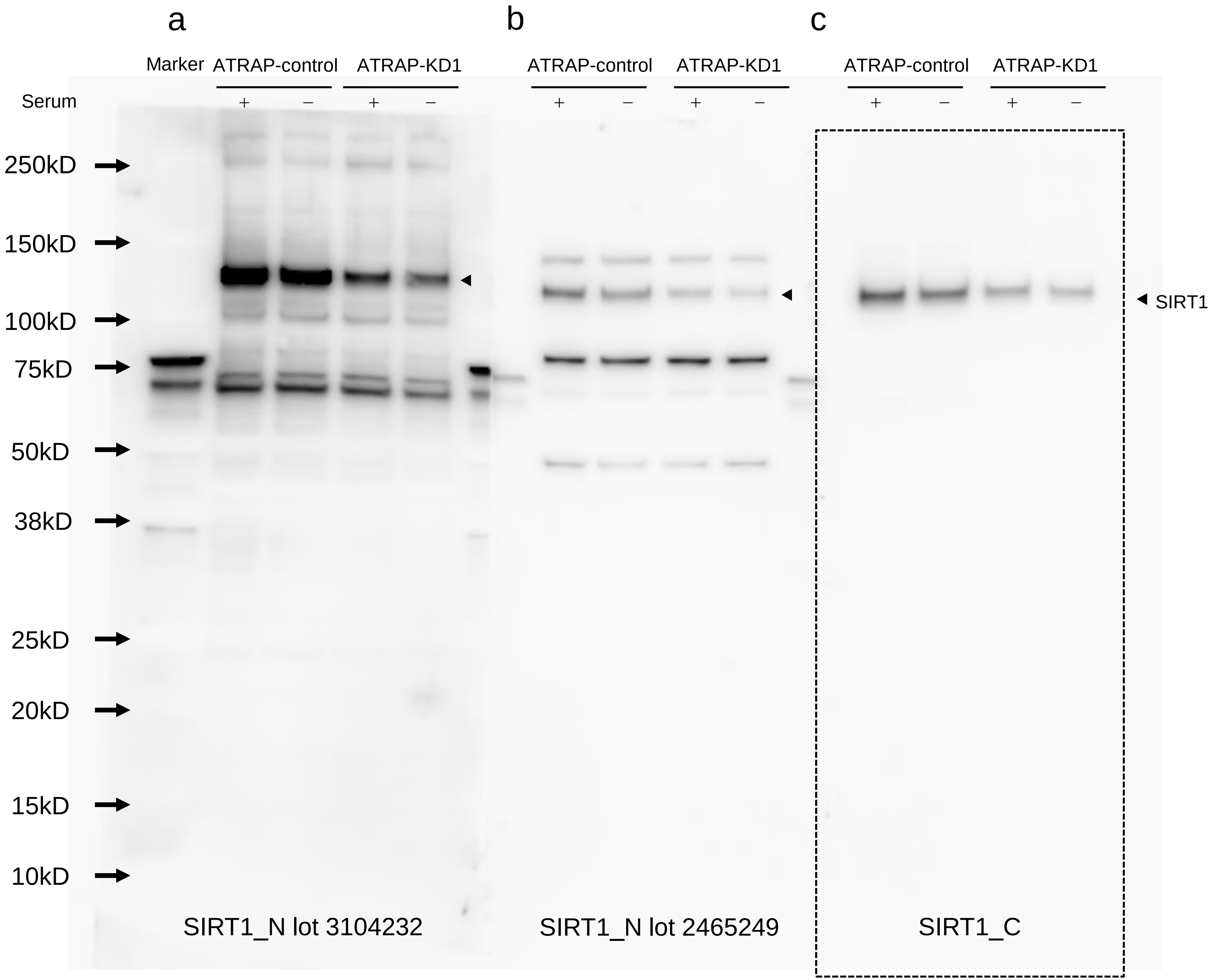

Supplemental Figure legend

FigureS3

Western blot analysis of alternative isoforms of SIRT1 protein detecting using antibodies targeted to N-terminal or C-terminal region of SIRT1.

a-c) Same samples of figure 4e were loaded. Membranes were probed with antibodies recognising N-terminal 131 amino acids (SIRT1_N_ lot 3104232 or _lot 2465249) or g C-terminal region (SIRT1_C). Arrow heads indicated SIRT1 protein isoform (NP_036370) that detected by all antibodies. Except NP_036370, no common signals were detected by SIRT1_N_ lot 3104232 and _lot 2465249. No expected molecular weight signals of alternative isoforms of SIRT1 protein (see figure S3) were detected by SIRT1_C.

FigureS4

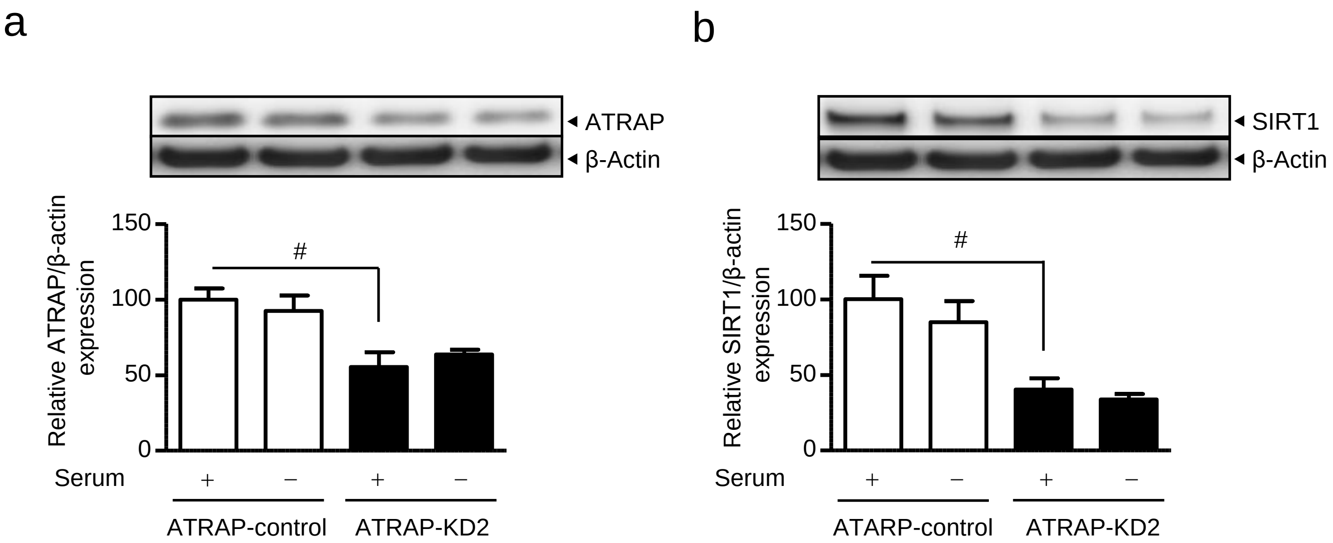
a
ATRAP
β-Actin
Relative ATRAP/β-actin expression
150
100
50
0
#
Serum
+
−
+
−
ATRAP-control
ATRAP-KD2
b
SIRT1
β-Actin
Relative SIRT1/β-actin expression
150
100
50
0
#
Serum
+
−
+
−
ATARP-control
ATRAP-KD2

Supplemental Figure legend

FigureS4

Effect of ATRAP knockdown on *SIRT1* mRNA and protein expression with or without serum-withdrawal.

The ciRPTEC were treated with negative control siRNA (ATRAP-control, S4a,b), ATRAP siRNA #2 (ATRAP-KD2, S4a and b) for 48 hours, followed by serum withdrawal for 24 hours. The relative protein expression of ATRAP and SIRT1 in the ciRPTEC was determined by western blot analysis, normalized to β-actin expression. Protein levels in the presence of serum (+) and control siRNA were set to 100. SIRT1 proteins were detected with an antibody towards the N-terminal 1–131 amino acids (SIRT1_N lot 2465249). All data were obtained with three biologically independent and were analysed by two-way ANOVA. Values represent the means ± standard error. a, b) #, $p < 0.05$ vs. ATRAP-control within the same serum groups.

FigureS5

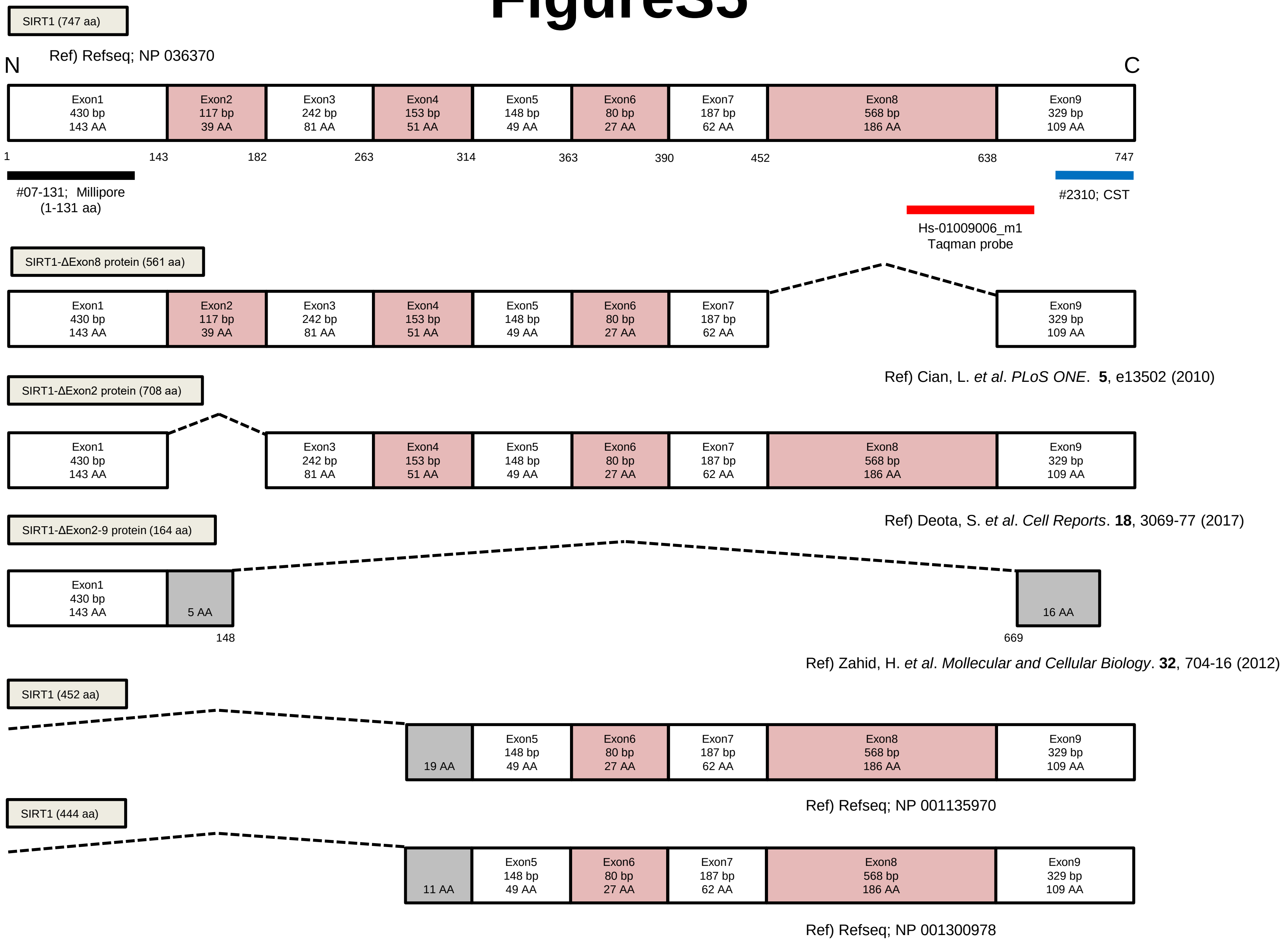
SIRT1 (747 aa)
Ref) Refseq; NP 036370
N
C
Exon1 430 bp 143 AA
Exon2 117 bp 39 AA
Exon3 242 bp 81 AA
Exon4 153 bp 51 AA
Exon5 148 bp 49 AA
Exon6 80 bp 27 AA
Exon7 187 bp 62 AA
Exon8 568 bp 186 AA
Exon9 329 bp 109 AA
1 143 182 263 314 363 390 452 638 747
#07-131; Millipore (1-131 aa)
#2310; CST
Hs-01009006_m1 Taqman probe
SIRT1-ΔExon8 protein (561 aa)
Exon1 430 bp 143 AA
Exon2 117 bp 39 AA
Exon3 242 bp 81 AA
Exon4 153 bp 51 AA
Exon5 148 bp 49 AA
Exon6 80 bp 27 AA
Exon7 187 bp 62 AA
Exon9 329 bp 109 AA
Ref) Cian, L. et al. PLoS ONE. 5, e13502 (2010)
SIRT1-ΔExon2 protein (708 aa)
Exon1 430 bp 143 AA
Exon3 242 bp 81 AA
Exon4 153 bp 51 AA
Exon5 148 bp 49 AA
Exon6 80 bp 27 AA
Exon7 187 bp 62 AA
Exon8 568 bp 186 AA
Exon9 329 bp 109 AA
Ref) Deota, S. et al. Cell Reports. 18, 3069-77 (2017)
SIRT1-ΔExon2-9 protein (164 aa)
Exon1 430 bp 143 AA
5 AA
16 AA
148
669
Ref) Zahid, H. et al. Mollecular and Cellular Biology. 32, 704-16 (2012)
SIRT1 (452 aa)
19 AA
Exon5 148 bp 49 AA
Exon6 80 bp 27 AA
Exon7 187 bp 62 AA
Exon8 568 bp 186 AA
Exon9 329 bp 109 AA
Ref) Refseq; NP 001135970
SIRT1 (444 aa)
11 AA
Exon5 148 bp 49 AA
Exon6 80 bp 27 AA
Exon7 187 bp 62 AA
Exon8 568 bp 186 AA
Exon9 329 bp 109 AA
Ref) Refseq; NP 001300978

Supplemental Figure legend

FigureS5

Schematic structures of alternative isoforms of SIRT1 protein.

White and pink boxes are indicated corresponding exon regions. Each boxes containing information of DNA length (bp) and amino acid length (AA). Gray boxes indicated isoform specific amino acids length (AA).

Black, blue, and red lines indicated SIRT1_N terminal 1-131 antigen, SIRT1_C terminal region antigen and corresponding mRNA region of Taqman probe targeting site.

Reference were showed on left side of schema of each isoform.

FigureS6

A whole gel of β-actin in Figure 4b,d

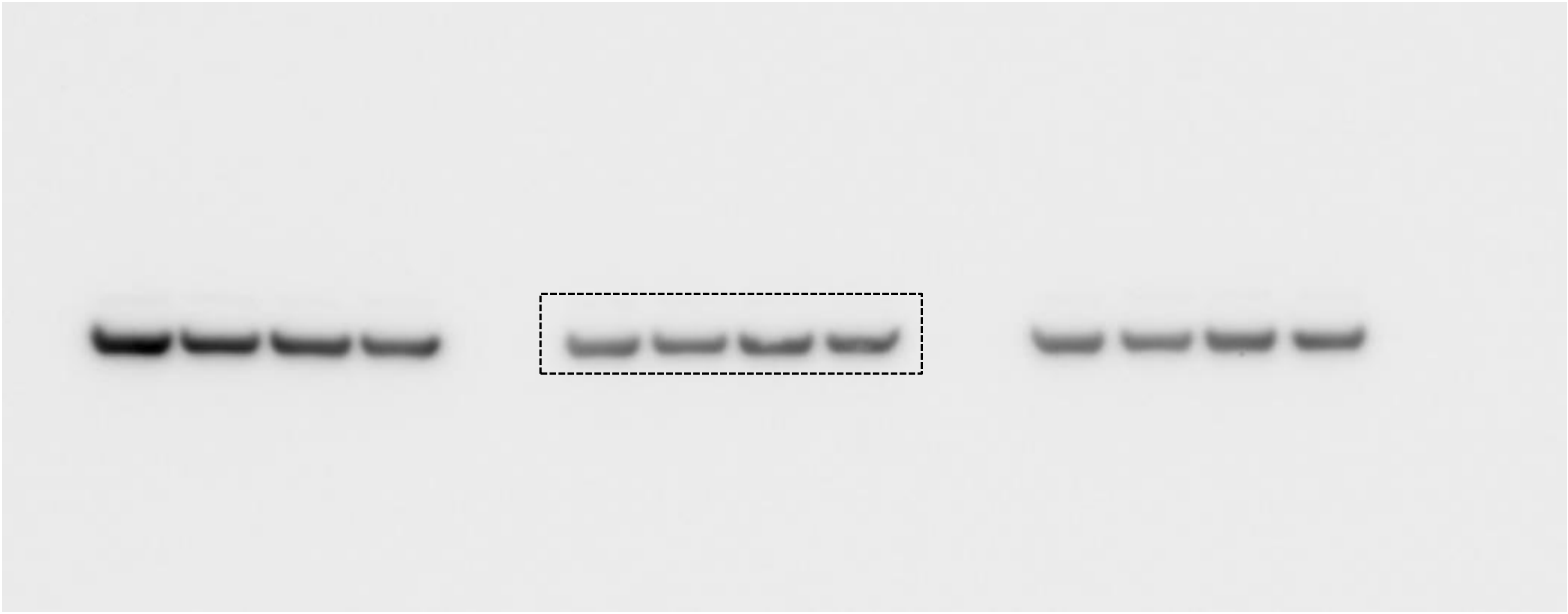

FigureS7

A whole gel of ATRAP in Figure 4b

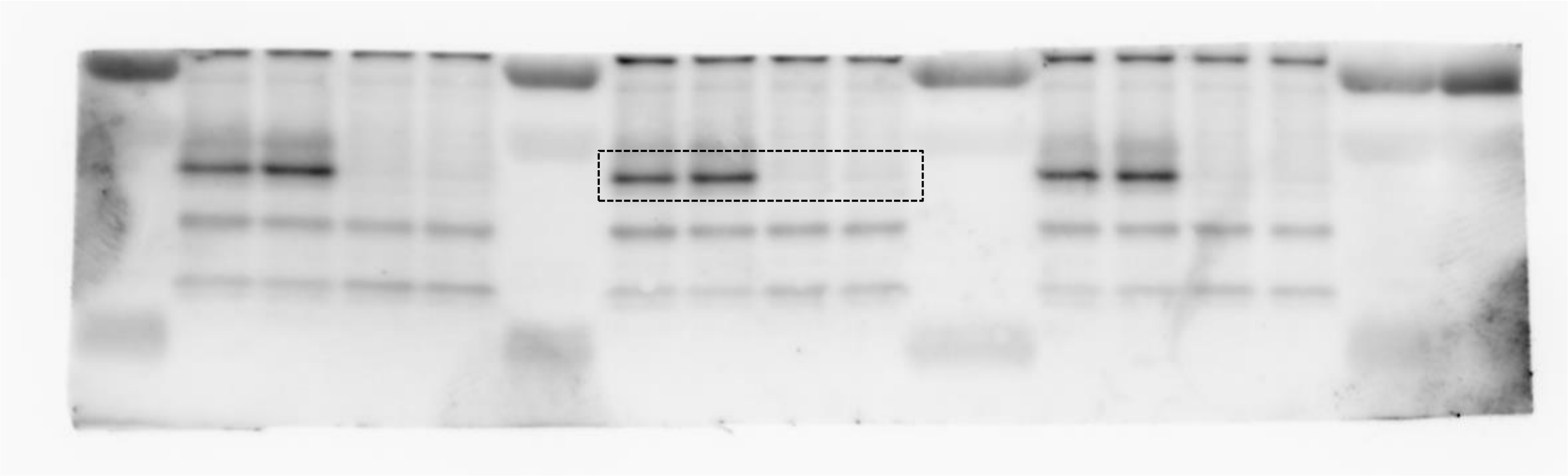

FigureS8

A whole gel of SIRT1 in Figure 4d

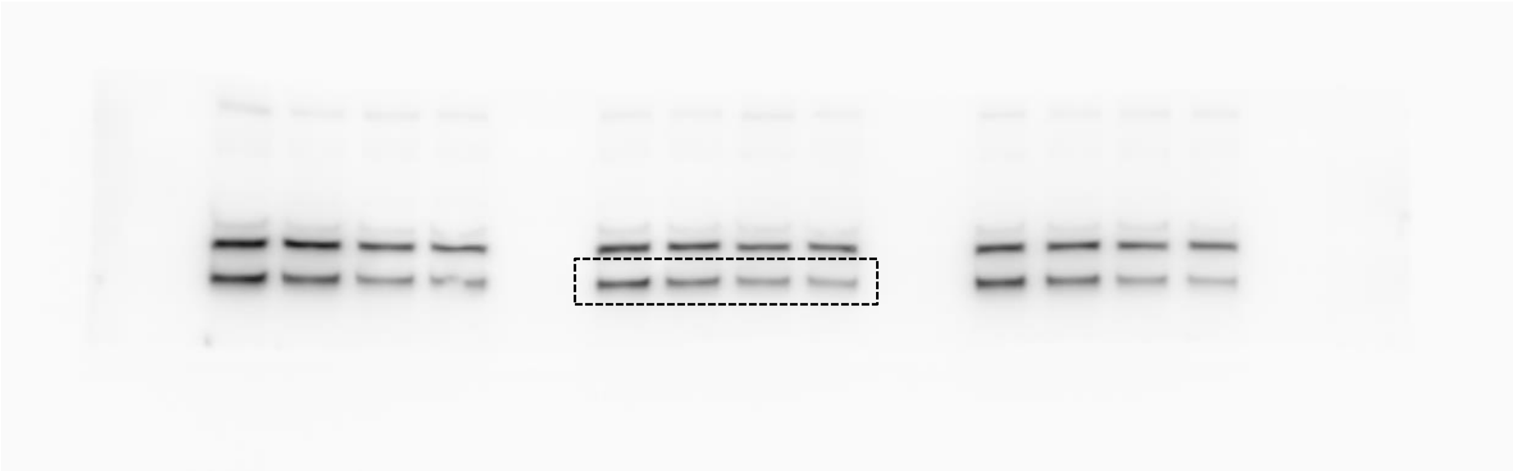

FigureS9

A whole gel of ATRAP in Figure 4f

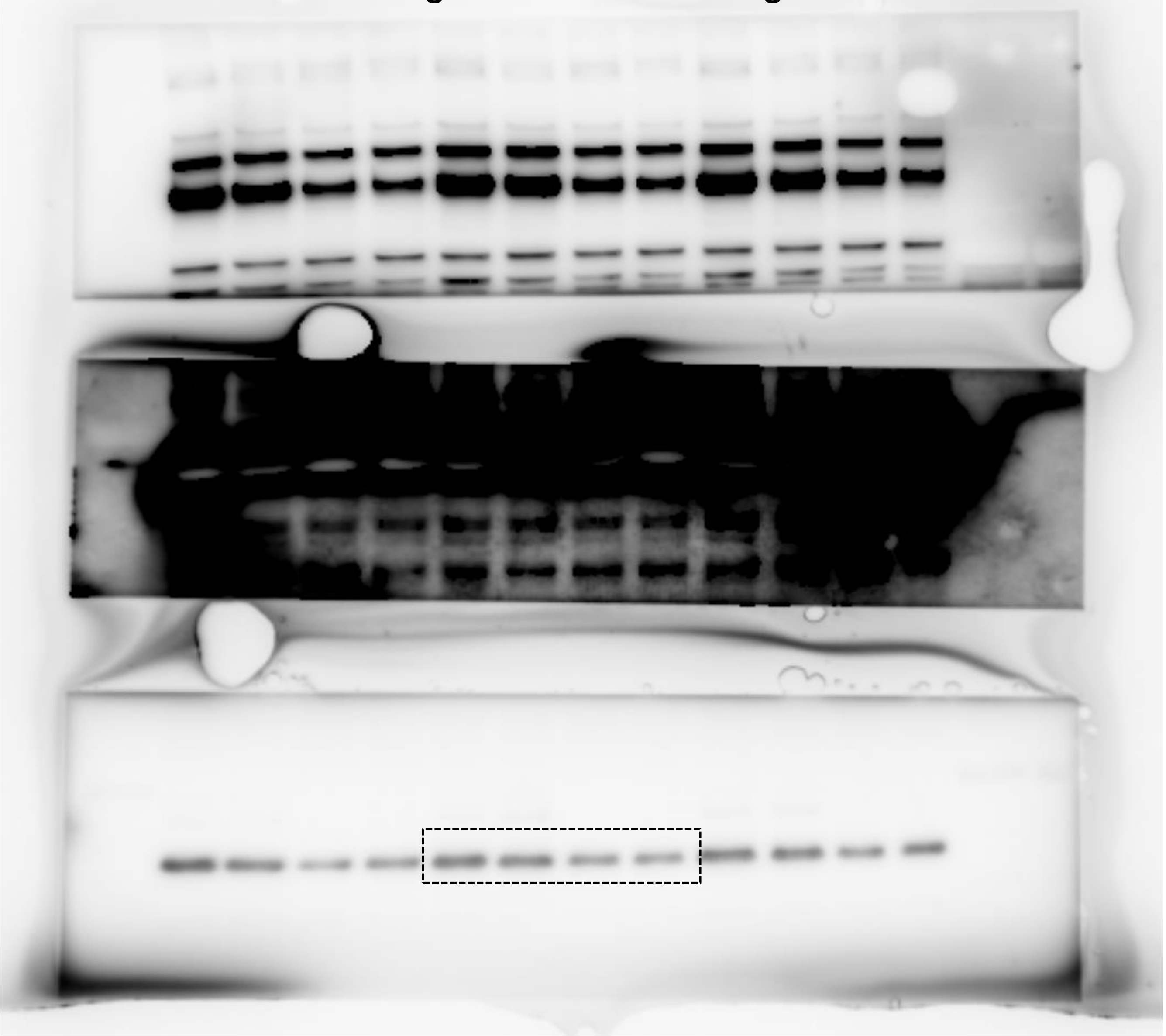

FigureS10

A whole gel of SIRT1 in Figure 4g

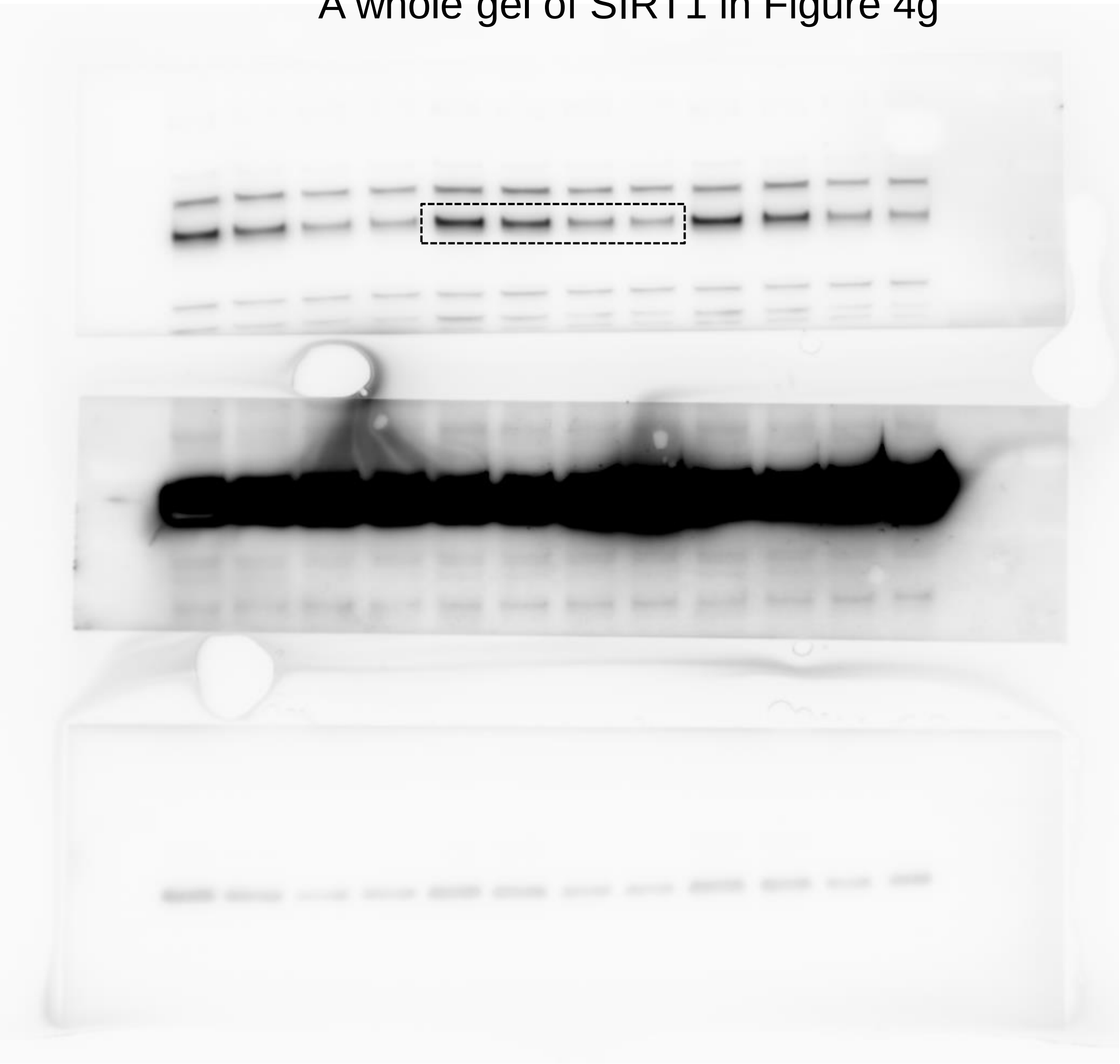

FigureS11

A whole gel of β-actin in Figure 4f,g

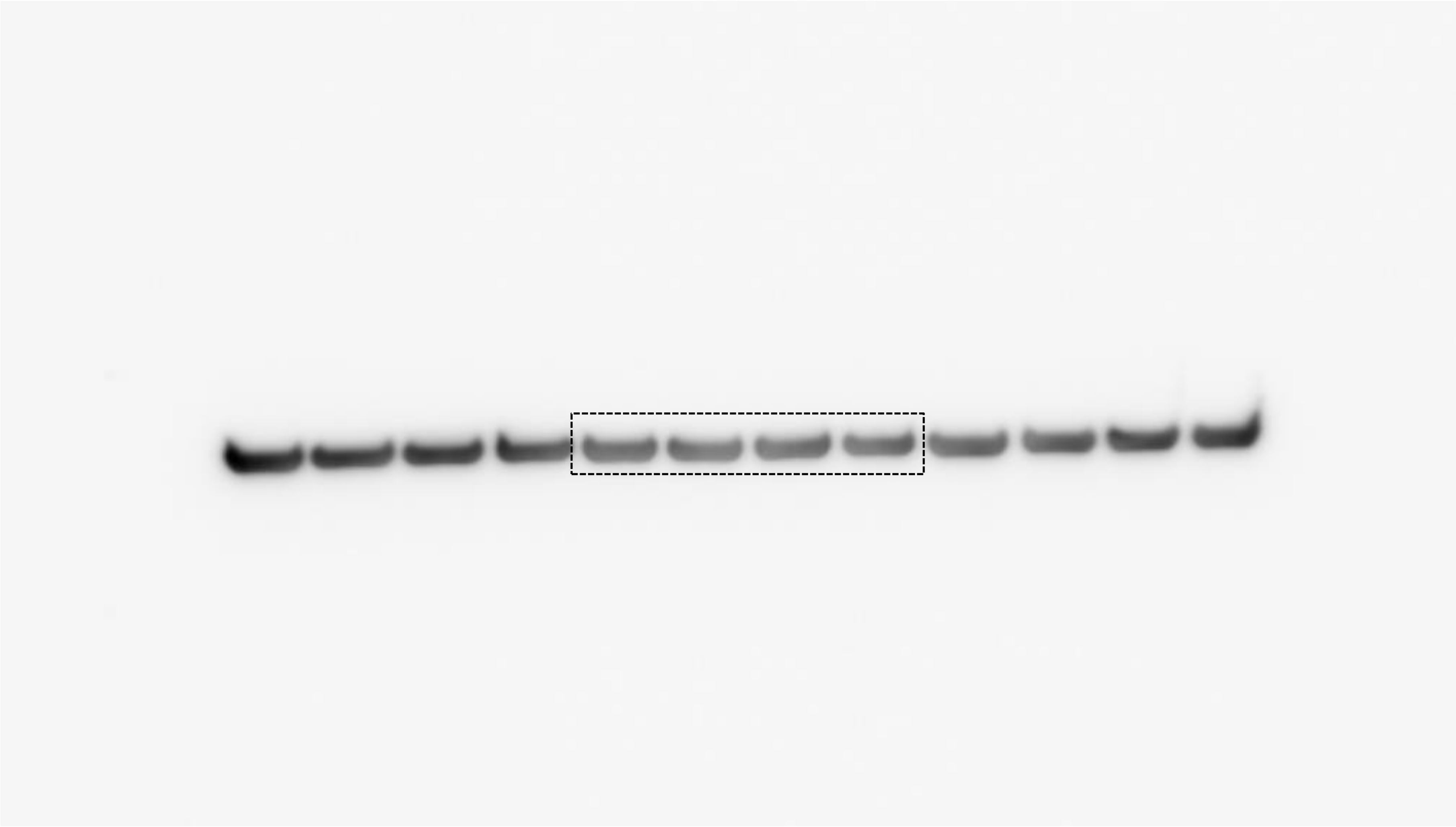

FigureS12

A whole gel of GAPDH, ATRAP and in SIRT1 Figure 4h

GAPDH

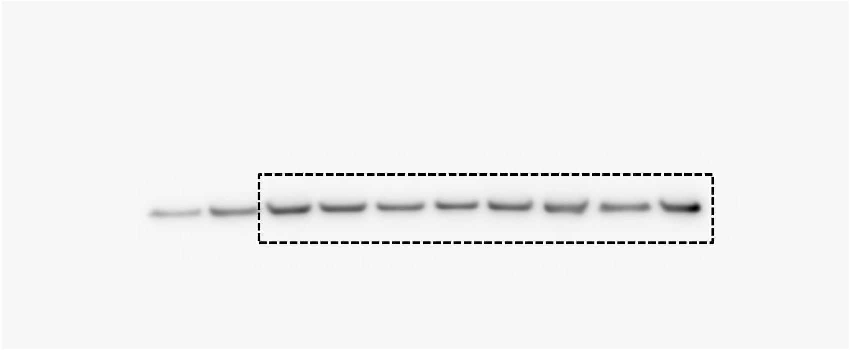

ATRAP

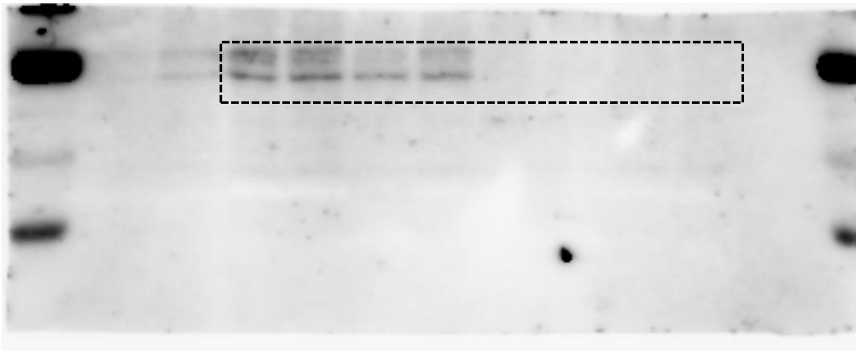

SIRT1 (short exposure)

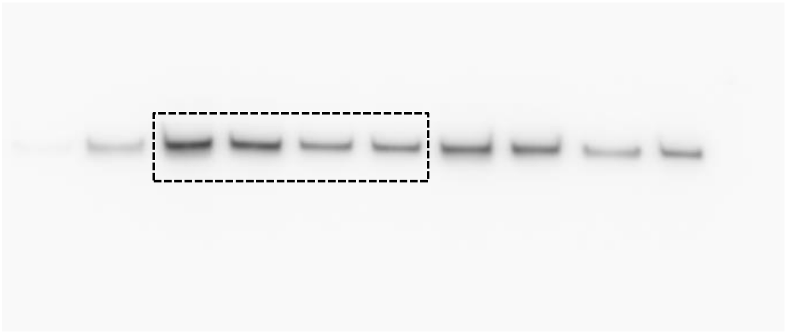

SIRT1 (long exposure)

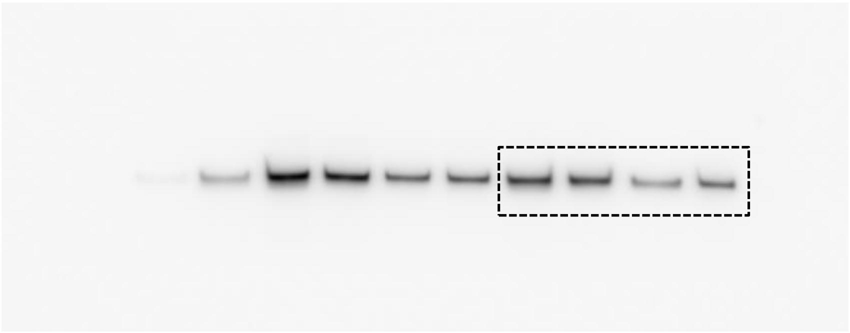

FigureS13

A whole gel of ATRAP in Figure 5a

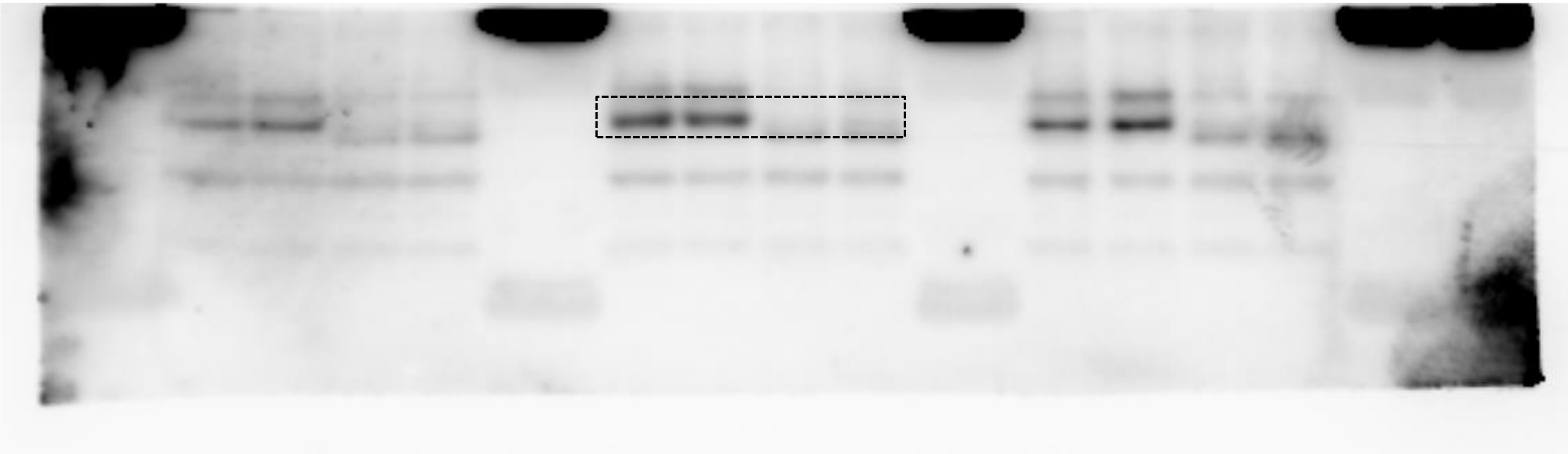

FigureS14

A whole gel of β-actin in Figure 5a,c

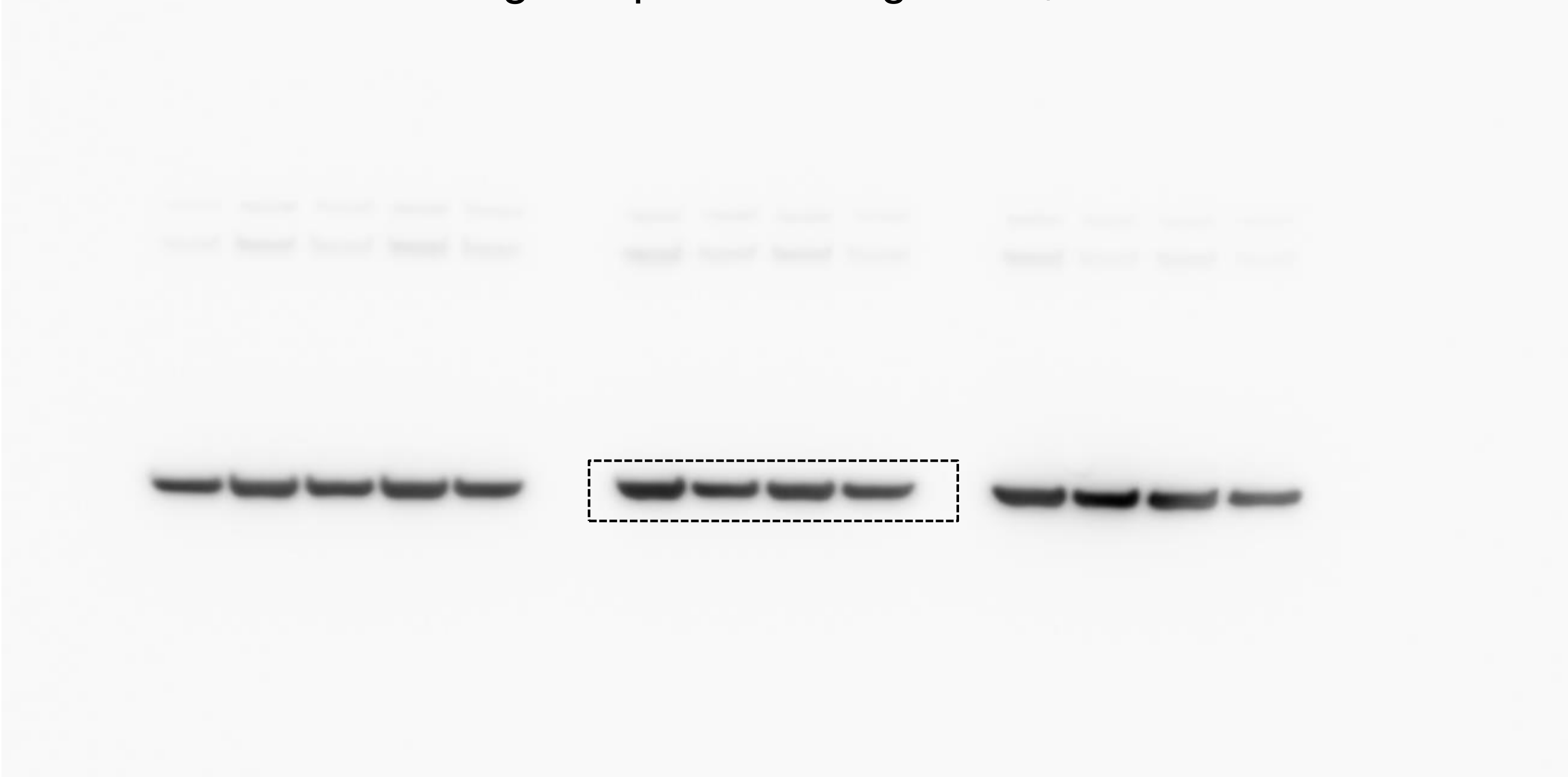

FigureS15

A whole gel of SIRT1 in Figure 5c

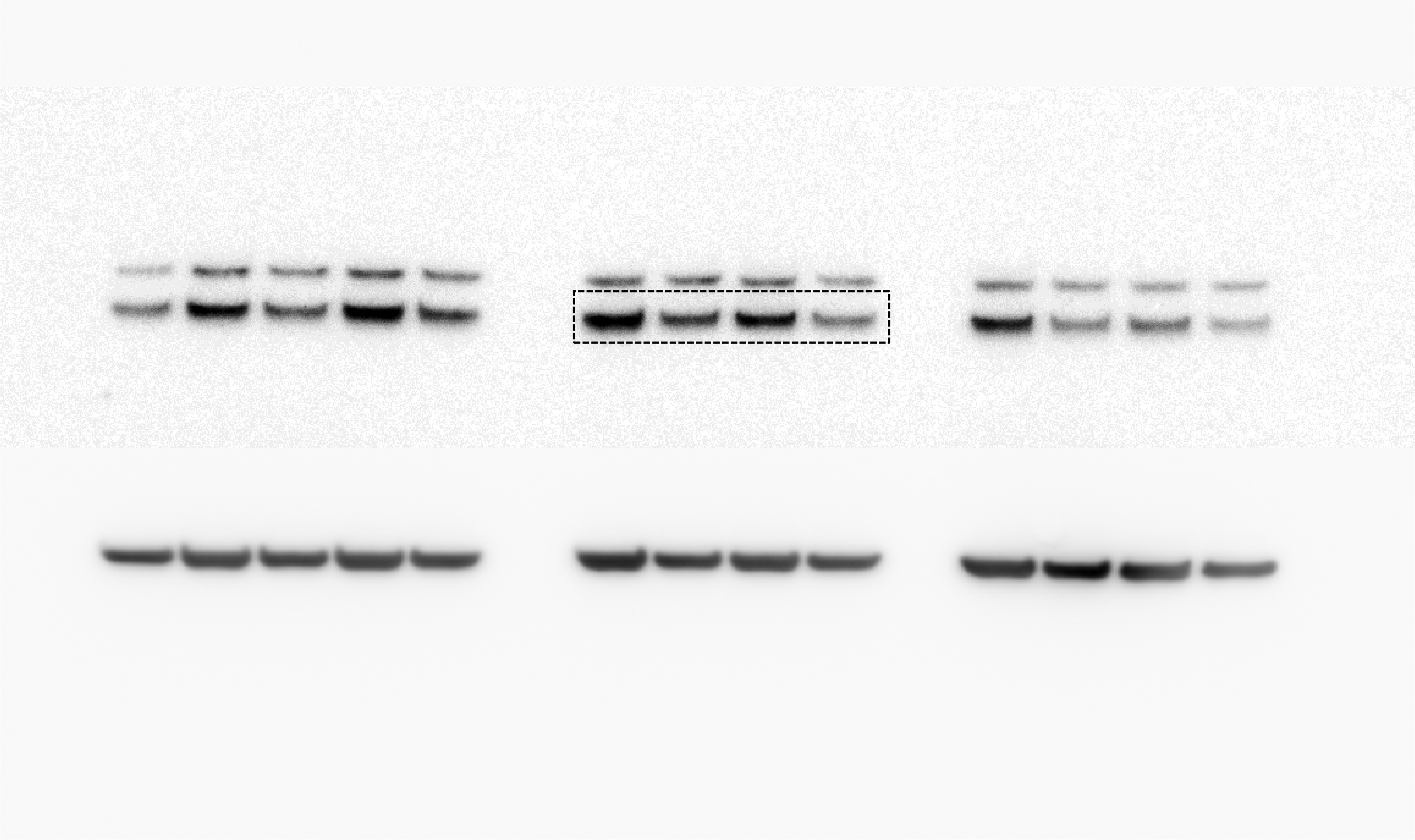

Supplement: Supplementary file 1 — Supplement figure [file 41598_2019_52566_MOESM1_ESM.pdf]
